# Evolutionary and functional characterization of lagomorph guanylate-binding proteins: a story of gain and loss and shedding light on expression, localization and innate immunity-related functions

**DOI:** 10.3389/fimmu.2024.1303089

**Published:** 2024-01-29

**Authors:** Luca Schelle, João Vasco Côrte-Real, Sharmeen Fayyaz, Augusto del Pozo Ben, Margarita Shnipova, Moritz Petersen, Rishikesh Lotke, Bhavna Menon, Dana Matzek, Lena Pfaff, Ana Pinheiro, João Pedro Marques, José Melo-Ferreira, Bastian Popper, Pedro José Esteves, Daniel Sauter, Joana Abrantes, Hanna-Mari Baldauf

**Affiliations:** ^1^ Max von Pettenkofer Institute and Gene Center, Virology, National Reference Center for Retroviruses, Faculty of Medicine, LMU München, Munich, Germany; ^2^ CIBIO-InBIO, Research Center in Biodiversity and Genetic Resources, University of Porto, Vairão, Portugal; ^3^ Department of Biology, Faculty of Sciences, University of Porto, Porto, Portugal; ^4^ BIOPOLIS Program in Genomics, Biodiversity and Land Planning, CIBIO, Vairão, Portugal; ^5^ Institute for Medical Virology and Epidemiology of Viral Diseases, University Hospital Tübingen, Tübingen, Germany; ^6^ National Institute of Virology, International Center of Chemical and Biological Sciences, University of Karachi, Karachi, Pakistan; ^7^ Biomedical Center (BMC), Core facility Animal Models (CAM), Faculty of Medicine, LMU München, Munich, Germany; ^8^ ISEM, University of Montpellier, CNRS, EPHE, IRD, Montpellier, France; ^9^ CITS - Center of Investigation in Health Technologies, CESPU, Gandra, Portugal

**Keywords:** GBP, evolution, innate immunity, antiviral proteins, cross-species conservation, lagomorphs, *Oryctolagus cuniculus*

## Abstract

Guanylate binding proteins (GBPs) are an evolutionarily ancient family of proteins that are widely distributed among eukaryotes. They belong to the dynamin superfamily of GTPases, and their expression can be partially induced by interferons (IFNs). GBPs are involved in the cell-autonomous innate immune response against bacterial, parasitic and viral infections. Evolutionary studies have shown that GBPs exhibit a pattern of gene gain and loss events, indicative for the birth-and-death model of evolution. Most species harbor large *GBP* gene clusters that encode multiple paralogs. Previous functional and in-depth evolutionary studies have mainly focused on murine and human GBPs. Since rabbits are another important model system for studying human diseases, we focus here on lagomorphs to broaden our understanding of the multifunctional GBP protein family by conducting evolutionary analyses and performing a molecular and functional characterization of rabbit GBPs. We observed that lagomorphs lack *GBP3, 6* and *7*. Furthermore, *Leporidae* experienced a loss of *GBP2*, a unique duplication of *GBP5* and a massive expansion of *GBP4*. Gene expression analysis by reverse transcriptase quantitative polymerase chain reaction (RT-qPCR) and transcriptome data revealed that leporid *GBP* expression varied across tissues. Overexpressed rabbit GBPs localized either uniformly and/or discretely to the cytoplasm and/or to the nucleus. *Oryctolagus cuniculus* (oc)GBP5L1 and rarely ocGBP5L2 were an exception, colocalizing with the trans-Golgi network (TGN). In addition, four ocGBPs were IFN-inducible and only ocGBP5L2 inhibited furin activity. In conclusion, from an evolutionary perspective, lagomorph GBPs experienced multiple gain and loss events, and the molecular and functional characteristics of ocGBP suggest a role in innate immunity.

## 1 Introduction

The survival of uni- and multicellular organisms depends on their ability to detect and eliminate invading pathogens (1), relying thereby on basic forms of immunity, such as Clustered Regularly Interspaced Short Palindromic Repeats (CRISPR) in bacteria, to complex immune systems in mammals (1). Upon infection, type I and type II IFN are produced, resulting in the expression of numerous IFN-stimulated genes (2). Several of these genes enhance the efficacy of cell-autonomous immunity (3, 4), including guanylate-binding proteins (GBPs), which are specialized for host defense against intracellular pathogens ranging from bacteria to viruses (3, 5).

The GBP family belongs to the large dynamin GTPase superfamily, which includes myxoma resistance (Mx) proteins, immunity-related GTPases, and the very large IFN-inducible GTPases. These proteins share structural and biochemical similarities such as the GTPase domain (6, 7). Mammalian GBP proteins vary in size from ~65 to 73 kDa and are mainly localized to the cytosol (5, 8). They possess a large GTPase domain at the N-terminus representing motifs for guanine nucleotide binding, specifically GxxxxGK and x(V/L)RD (9–13), followed by a middle domain and the GTPase effector domain at the C-terminus (14). Human GBP1, 2 and 5 also harbor a CaaX motif at the C-terminus, which is important for isoprenylation and enables membrane anchoring (14).

The human genome encodes seven *GBPs* (*GBP1-7*) in a single cell cluster (15). It has been described that each *GBP* originated from the same common ancestor. Following the first duplication round, one gene evolved a CaaX motif, giving origin to modern day human *GBP1/2/3/5*. The second gene gave rise to human *GBP4/6/7*, which are characterized by the L182V replacement in the GTP-binding motif (TLRD) (15). *GBP1*, *2* and 3 are closely related members, with human *GBP1* and *3* sharing 87% amino acid similarities, while human *GBP2* shares 77% and 76% identity with human *GBP1* and *3*, respectively (15). On the other *GBP* branch, the most closely related genes are *GBP4* and *GBP7*, sharing 81% identity (15). We have recently studied the evolution of GBPs in primates (16) and found that *GBP3* evolved from a duplication of *GBP1* only in *Simiiformes*, while the duplication of *GBP4* gave rise to *GBP7*, which is only present in primates (16). In contrast, *GBP4* and *GBP5* are no longer present in the genomes of Old World monkeys (16). We have further extended evolutionary analyses to muroid *GBPs*, which are separated into two gene clusters and proposed a new nomenclature, as primate *GBP1, GBP3* and *GBP7* are absent from muroid genomes (17). In contrast, murine *Gbp2*, *Gbp5* and *Gbp6* might be true orthologs of their primate counterparts. Orthologs are genes in different species that evolved from a common ancestral gene through speciation and may retain the same function throughout evolution. Identification of orthologs is a critical process for reliable prediction of gene function in newly sequenced genomes. More importantly, four *Gbps* are exclusive to muroids, but absent from *Mus musculus* (17). Thus, in line with the proposed birth-and-death model of evolution, our analyses revealed that GBPs underwent duplications, deletions, and neofunctionalizations, raising even more awareness to conduct in-depth evolutionary analyses for GBPs of different species. Beyond primates and muroids, information on the evolution and function of GBPs is scarce. In addition to humans, the role of GBPs in innate immunity has been described in plants, invertebrates, teleosts, mice, pigs, and *Tupaia* (14).

Within Lagomorpha, there are two families, *Leporidae* (hares and rabbits) and *Ochotonidae* (pikas), which diverged approximately ~37 million years ago (MYA) (18). The *Ochotonidae* family is restricted to the genus *Ochotona*, which is further divided into four subgenera (*Pika, Logotona, Conothoa* and *Ochotona*) and the divergence time between these subgenera is ~7 to 14 MYA (13, 19–21). The *Leporidae* family is divided into two groups, hares and rabbits, which diverged around 12 MYA (22). The hare group only contains one genus, *Lepus*, while the rabbit group comprises ten distinct genera (23, 24). The genus *Oryctolagus* is one of the most studied due to its importance in the Mediterranean ecosystem as prey for endangered species and also for its importance in biomedical research, particularly in immunology and infectious diseases (24, 25). Furthermore, the genetic diversity of innate immunity genes between rabbits and humans is lower than between mice and humans, suggesting that the European rabbit might be a better model to study such genes (26).

In this study, we aimed to characterize the evolutionary history and intrinsic functions of lagomorph GBPs, going beyond their description in murines and primates, to broaden the understanding of the GPB family. For this, we combined evolutionary analyses with *in vitro* assays, shedding light on species-specific mRNA and protein expression profiles and evolutionary patterns. In addition, we wanted to establish links to cell-autonomous innate immunity functions of GBPs.

## 2 Results

### 2.1 Absence of *GBP3/6/7* in lagomorphs; loss of *GBP2*, unique duplication of *GBP5* and expansion of *GBP4s* in leporids

We analyzed 204 *GBP* sequences belonging to muroids, primates, lagomorphs, *Tupaia*, elephant and chicken. Before conducting the evolutionary analysis, the *GBP*s alignment (see **Supplementary Table 1** for accession numbers and see **Supplementary Data** for *GBP* alignment) was screened for recombination and gene conversion using GARD (Genetic Algorithm for Recombination Detection; 27). No gene conversion or recombination events were detected (data not shown). Thirty-one sequences were excluded because they did not encode a functional protein or the sequence was truncated (see **Supplementary Table 1** for accession numbers).

**Figure 1:**
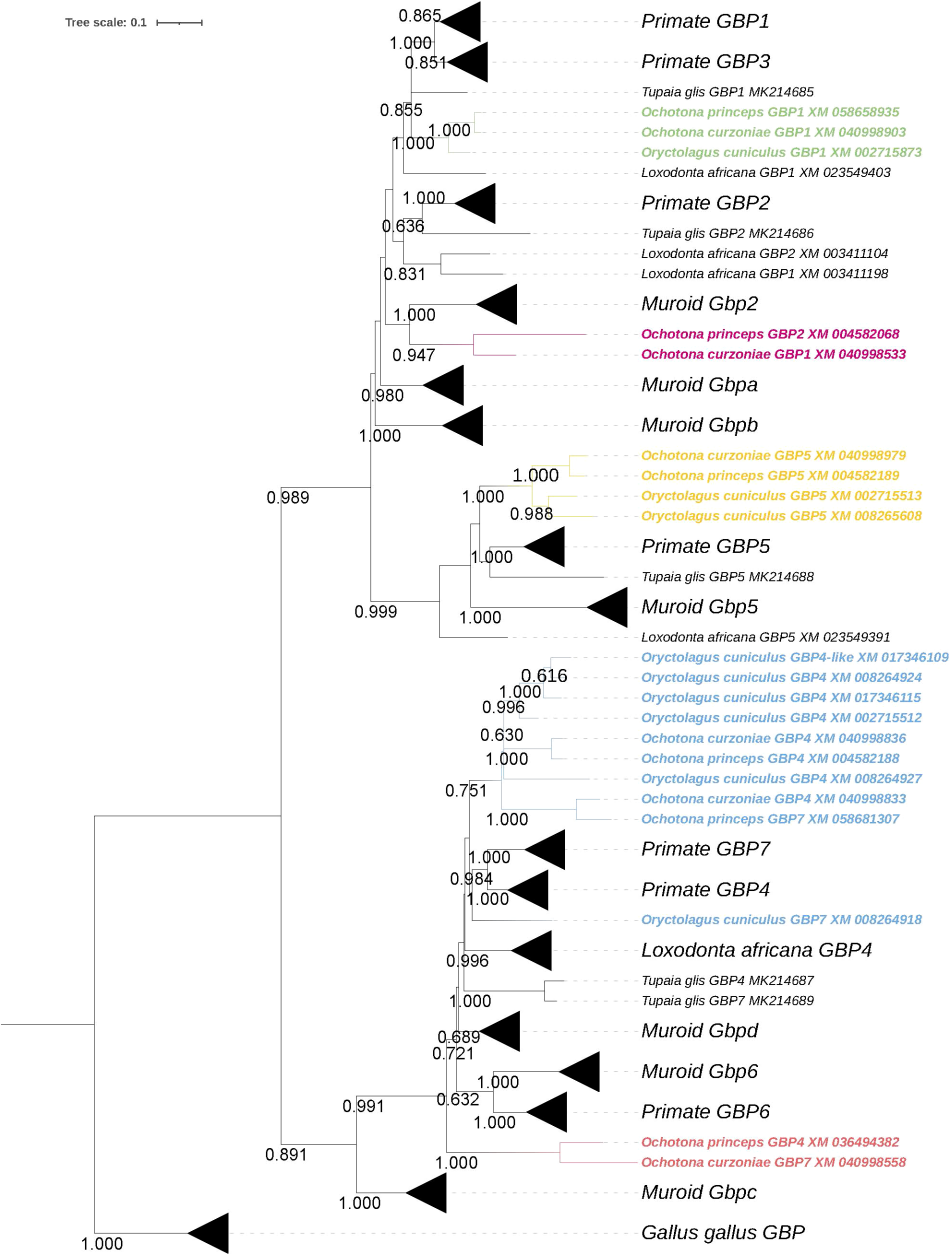
Phylogeny of lagomorph *GBPs*. The evolutionary history was inferred by using the Maximum Likelihood (ML) method. The tree is drawn to scale, with branch lengths measured in the number of substitutions per site. The bootstrap values are shown next to the branches and only values >0.6 are shown (iTOL was used for tree visualization; 28).

**Figure 2:**
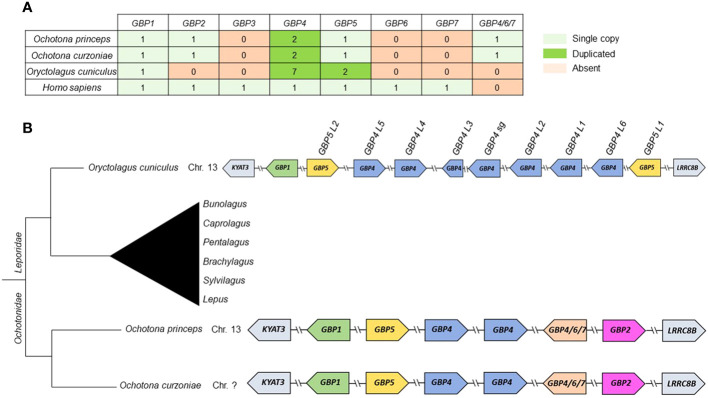
Summary of gain/loss and synteny of lagomorph *GBPs*. **(A)** Number of *GBP* copies for each lagomorph species. **(B)** Gene synteny of lagomorph GBPs. The gene synteny of the lagomorph GBPs is displayed for the analyzed lagomorph species (right). GBPs are colored following the grouping in the phylogenetic analyses (**Figure 1**). Arrows indicate gene orientation. Lagomorph phylogeny is shown (left) and the diagram is not to scale.

The ML phylogenetic tree showed that lagomorphs do not have *GBP3*, *6* and *7*, as none of the sequences grouped with the corresponding human counterpart (**Figure 1**). *Ochotonidae* appear to harbor one copy of *GBP2* in their genome (**Figure 1**). Interestingly, *GBP2* is not present in *Oryctolagus cuniculus* nor could be found in the genome of *Lepus* (data not shown), indicating that *GBP2* was lost in the ancestor of *Leporidae* at least 12 MYA (22). Moreover, lagomorphs diverged from the common ancestor with rodents about 62-100 MYA (29, 30), which may explain why muroid *Gbp2* and *Ochotonidae GBP2* cluster together and not with primate *GBP2* despite the low bootstrap value (<0.6) (**Figure 1**). This group was named as *Ochotonidae GBP2* because in a previous study, muroid *Gbp2* clustered with primate *GBP2* (17). A summary of the gain and loss of *GBPs* in lagomorph is presented in **Figure 2A**. *GBP1* is present in *Leporidae* and *Ochotonidae*, with one copy in each species, similar to primates (**Figure 1**). The *GBP5* cluster was extremely robust with a bootstrap value of 1.00 (**Figure 1**). Lagomorph *GBP5* was present in all species with *Ochotonidae* having only one copy, whereas *Oryctolagus cuniculus* had two copies. Moreover, this duplication was also present in *Lepus* (data not shown), suggesting a duplication of *GBP5* after the split of *Ochotonidae* and *Leporidae* and before the split of *Lepus* and *Oryctolagus* (~12 MYA; 22; ~37 Mya; 18). A major cluster, designated as *GBP4*, underwent an expansion in *Oryctolagus cuniculus* with seven copies of the gene (*GBP4 XM_017345575* was not included in the analysis) (**Figure 1**), while *Ochotona curzoniae* and *princeps* presented two copies. From Maximum Likelihood (ML) tree, *Oryctolagus cuniculus GBP7* did not cluster with lagomorph *GBP4* but was at a basal position of the cluster of primate *GBP4* and *7*. However, the low bootstrap value (<0.6) indicated that the phylogenetic relationship could not be fully resolved. Despite this, the nomenclature of this gene might be incorrect since *GBP7* is only present in primates (16, 17) and it did not cluster with primate *GBP7* in the ML tree (**Figure 1**). As such, we designated it *ocGBP4*; however, throughout the manuscript we named it *ocGBP4L6* (locus 6). No *GBP6* could be found in lagomorphs, as no lagomorph *GBP* clustered with primate and muroid *GBP6* (**Figure 1**). The most likely explanation is that *GBP6* was deleted from the lagomorph genome after the split from rodents since it is present in rodents. One might speculate that the expansion of *GBP4* in lagomorphs could be a compensation mechanism for the loss of *GBP6*. Interestingly, a group with *GBP* sequences from both *Ochotona* species was found at a basal position from the *GBP4*, *6* and *7* group (**Figure 1**). The origin of this group was puzzling, and it could be explained by a duplication event of the ancestral gene of *GBP4/6/7* originating from this group in *Ochotonidae* which then underwent an accelerated mutation rate. We designated this group as *GBP4/6/7* (**Figure 1**). Based on the evolutionary analysis, we suggest a new nomenclature for genes that appeared to be misclassified (see **Table 1**).

**Table 1:** Protein sequence motifs of ocGBPs.

ProSite Identifier	PS51715|G_GB1_RHD3	PS50313|GLU_RICH	PS50079|NLS_BP
Motif	GB1/RHD3-type guanine nucleotide-binding (G) domain	Glutamic acid enriched region	Bipartite nuclear localization signal
**ocGBP1**	Yes (high conf.)	No	Yes (2x, low conf.)
**ocGBP4L1**	Yes (high conf.)	Yes (low conf.)	Yes (2x, low conf.)
**ocGBP4L2**	Yes (high conf.)	Yes (low conf.)	Yes (1x, low conf.)
**ocGBP4L3**	Yes (high conf.)	No	No
**ocGBP4L4**	Yes (high conf.)	Yes (high conf.)	Yes (1x, low conf.)
**ocGBP4L5**	Yes (high conf.)	Yes (low conf.)	Yes (1x, low conf.)
**ocGBP4sg**	Yes (high conf.)	Yes (low conf.)	Yes (1x, low conf.)
**ocGBP5L1**	Yes (high conf.)	No	No
**ocGBP5L2**	Yes (high conf.)	Yes (low conf.)	No
**ocGBP4L6**	Yes (high conf.)	No	Yes (1x, low conf.)

conf., confidence.

Considering the synteny of the lagomorph *GBP* genes, the gene cluster was located in a single chromosome, similar to primates (15, 16) (**Figure 2B**). Both *Ochotona* species presented the same synteny (**Figure 2B**). In all three lagomorph species, the *GBP* gene cluster was flanked by *KYAT3* and *LRRC8B*, as described elsewhere (16, 17). In conclusion, lagomorph *GBP* genes showed patterns of gain and loss and shared similarities with primate and muroid *GBPs*. However, they evolved independently after the separation from other mammals.

### 2.2 Conserved *GBP-*specific motifs in the lagomorphs

In order to shed light on the protein structure of lagomorph GBPs, we analyzed GBP-specific motifs. Except for *Ochotona curzoniae* (XM_ 040998558), all *GBPs* share a GxxxxGK guanine nucleotide binding motif. The TLRD/TVRD motif, important for guanine base contact, is present in all *GBPs*, except for *Oryctolagus cuniculus (oc)GBP4 L3* (XM_017345575), which encodes a truncated GBP with only 129 amino acids (see **Supplementary Table 2**; see *GBP* alignment, **Supplementary Data**). Most of the *GBPs* in the main *GBP1*/*2*/*5* cluster possess a TLRD motif instead of a TVRD motif (15). We observed that lagomorph *GBPs* from the *GBP1/2/5* group contain the TLRD motif, while *GBP2* from *Ochotona princeps* (XM_004582068) harbors an AVRD motif instead (see **Supplementary Table 2**). Lagomorph *GBPs* from the major *GBP4*/*6*/*7* cluster possess a TVRD or an AVRD motif. An exchange of a threonine for an alanine has also been observed in rodents (9). Interestingly, *ocGBP4 L6* (XM_008264918) carries a cysteine instead of a threonine (CVRD) (see **Supplementary Table 2**). In summary, lagomorph GBPs have in general similar guanine nucleotide binding motifs and motifs for guanine base contact as described for other mammalian GBPs.

### 2.3 Presence of different motifs with high probability of occurrence with a phylogeny-specific prenylation motif

As the European rabbit (*Oryctolagus cuniculus*) has been widely used as an animal model in biomedical research, we focused on the analysis of ocGBPs. We analyzed the ocGBP sequences for protein sequence motifs using the ProSite Scan tool (31–33). The results of the analysis (**Supplementary Table 2**) are summarized in **Table 1** (Protein sequence motifs) and **Table 2** (Protein sequence motifs with a high probability of occurrence). We observed that the G domain was the only conserved motif that was predicted with high confidence. With low confidence, the C-terminal glutamic acid-rich and nuclear localization signals were also found in the majority of analyzed ocGBPs (**Table 1**). In addition, protein sequence motifs were predicted with high occurrence, including sites of N-glycosylation, phosphorylation, ATP/GTP-binding motifs (P-loops), amidation, and N-myristyolation, which were found in varying numbers in the analyzed GBPs (for location, number and sequence motif see **Supplementary Table 3**). In all analyzed rabbit GBPs, the conserved P-loops were in accordance with the conserved G domain. Furthermore, prenyl group binding sites (CaaX motifs) were found only at the C-termini of ocGBP1 and ocGBP5 L2 (**Table 2**). However, we cannot rule out that alternative splicing might occur in rabbit GBPs and that it could impact some important motifs and dysregulate function. In summary, ocGBP paralogs have acquired individual protein sequence motifs but shared a highly conserved G domain and similar putative post-translational modification sites (PTMs).

**Table 2:** Protein sequence motifs with a high probability of occurrence.

ProSite Identifier	PS00004|CAMP_PHOSPHO_SITE PS00005|PKC_PHOSPHO_SITE PS00006|CK2_PHOSPHO_SITE PS60007|TYR_PHOSPHO_SITE_2	PS00017|ATP_GTP_A	PS00009|AMIDATION	PS00008|MYRISTYL	PS00294|PRENYLATION
Motif	Phosphorylation sites	ATP/GTP-binding site motif A (P-loop)	Amidation site	N-myristoylation site	Prenyl group binding site (CAAX box)
**ocGBP1**	Yes (22x)	Yes	Yes	Yes (3x)	Yes (CVIS)
**ocGBP4L1**	Yes (18x)	Yes	Yes	Yes (6x)	No
**ocGBP4L2**	Yes (14x)	Yes	Yes	Yes (9x)	No
**ocGBP4L3**	Yes (3x)	Yes	No	Yes (2x)	No
**ocGBP4L4**	Yes (20x)	Yes	No	Yes (8x)	No
**ocGBP4L5**	Yes (17x)	Yes	No	Yes (8x)	No
**ocGBP4sg**	Yes (15x)	Yes	No	Yes (10x)	No
**ocGBP5L1**	Yes (14x)	Yes	No	Yes (3x)	No
**ocGBP5L2**	Yes (18x)	Yes	No	Yes (3x)	Yes (CILL)
**ocGBP4L6**	Yes (19x)	Yes	Yes	Yes (4x)	No

### 2.4 Conserved predicted tertiary structure of ocGBPs among the phylogenetic subgroups

**Figure 3:**
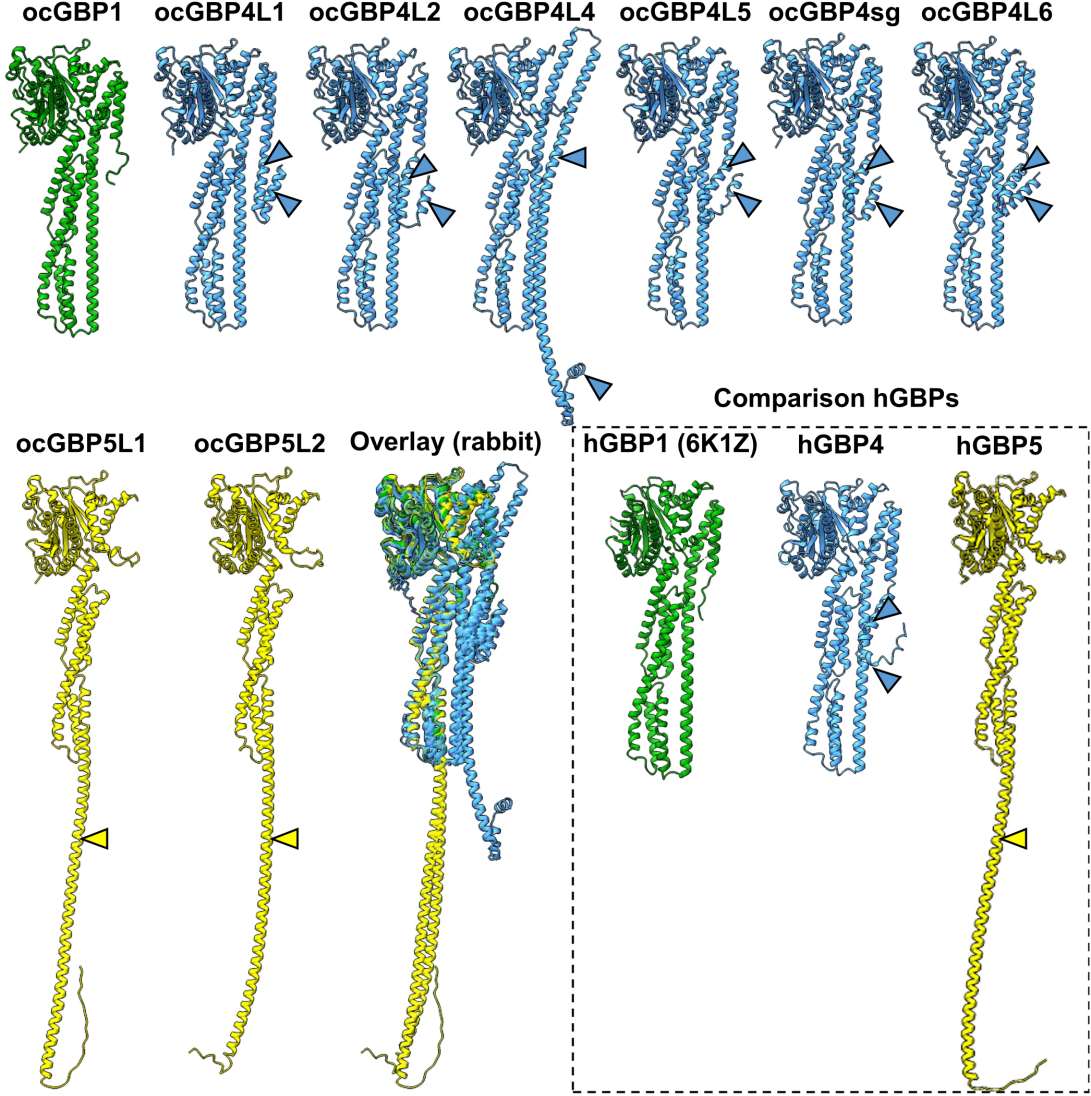
Prediction of ocGBP tertiary structures. Best predicted model per GBP is shown. Rabbit GBPs structures were predicted using AlphaFold. For comparison, as comparison structural data of hGBP1 (PDB: 6K1Z) and the predictions for hGBP4 and hGBP5 are also shown. GBPs are colored following the grouping in the phylogenetic analyses (**Figure 1**).

Since structural data are available only for human GBPs, the tertiary structure of ocGBPs was predicted using AlphaFold (**Figure 3**). ocGBP4 L3 was excluded due to its length. We found that all ocGBPs shared a similar structure with hGBP1/2 and hGBP5, which have been crystallized without GTPase effector domain (GED) (PDB accession numbers: 6K1Z, 7E58, 7E59). ocGBP1 appeared to have the same architecture as hGBP1 (**Figure 3**). For ocGBP4L1/L2/L4/L5/sg/L6, we observed two additional short α-helices at the C-terminus (blue arrow in **Figure 3**), with ocGBP4L4 having an extended α13 helix (blue arrows in **Figure 3**). For ocGBP5, the large globular domain (LGD) and the middle domain (MD) appeared to be similar to those of hGBP5. The GED was predicted as an elongated α-helix in an “open” state conformation (yellow arrow **Figure 3**), as proposed for the active conformation of hGBP1 (34–38). In conclusion, the structure of the GBPs seem to be highly conserved in the LGDs and MDs, while the GED is variable between phylogenetic groups but specific within them.

### 2.5 Varying endogenous expression levels of ocGBPs

**Figure 4:**
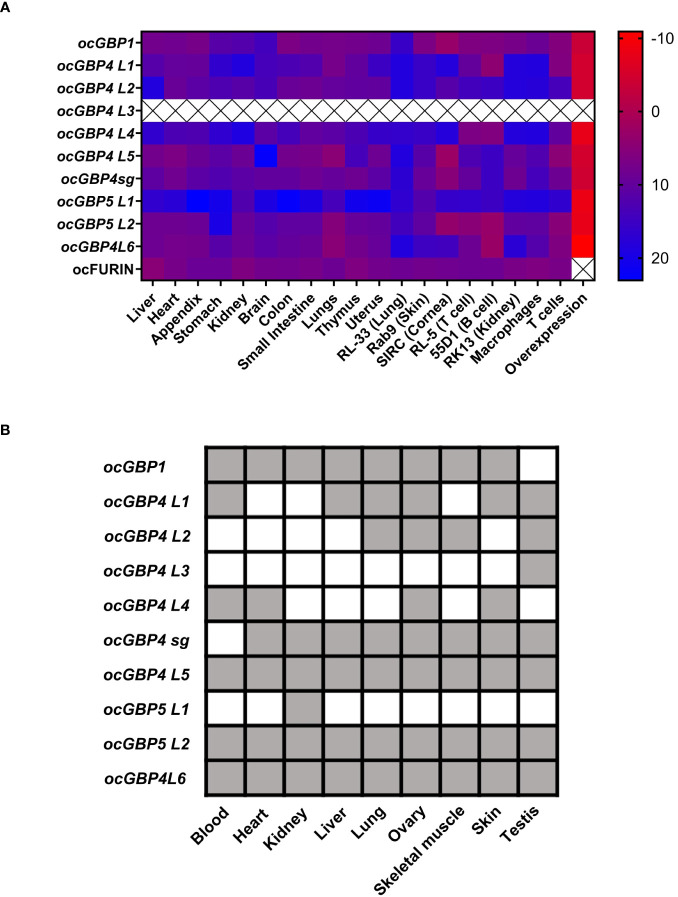
Differential mRNA expression levels for rabbit GBPs. **(A)** Heat map of RT-qPCR mRNA expression analysis of ocGBPs and ocFurin in several tissues, primary cells, cell lines and overexpression in RK13 cells: ΔCt values to the reference gene *ActinB* are displayed (CtGBP – CtActB). Tissues of four female New Zealand white rabbits and three primary cells, cell lines and overexpression were analyzed. Scale: from red (low ΔCt value, i.e., higher expression of target gene) to blue (higher ΔCt, i.e., lower expression of target gene). **(B)** Rabbit transcriptome was retrieved from (39) and blasted for *GBP* mRNA expression using the BLAST tool from NCBI. Gray color means present, white means absent.

To gain more insight into ocGPBs, we examined their gene expression profiles. We established and validated RT-qPCRs for ocGBPs (data not shown) and ocFurin as control, and analyzed mRNA levels in various rabbit tissues, primary cells and cell lines, including overexpression of ocGBPs in the rabbit kidney cell line (RK13 cells; **Figure 4A**). We also analyzed the transcriptome of *Oryctolagus cuniculus* for the presence of the ocGBPs (**Figure 4B**) ocFurin was ubiquitously expressed in all samples analyzed. We detected a distinct pattern of GBP expression levels. mRNA levels for *ocGBP4L1/4L2/4L4/5L1* were lower in most tissues, primary cells and cell lines examined than those of *ocGBP1/4L3/4L5/4sg/5L2/4L6*. In comparison, *ocGBP5L1* only showed higher expression in lung and kidney tissues and in the rabbit skin fibroblast cell line Rab9. On average, *ocGBP1/4L3/4sg/4L5/5L2/4L6* were 76-fold more expressed compared to the low expressors (**Figure 4A**). These results were largely consistent with the transcriptome data, where *ocGBP1/4L5/4sg/5L2/4L6* transcripts were also present in most of the tissues examined, and a higher number of tissues lacked detectable expression of *ocGBP4L1/4L2/4L4/5L1* (**Figure 4B**). Notably, *ocGBP4L3* mRNA was only found in the testis in the transcriptome data, whereas the RT-qPCR data showed expression comparable to other GBPs tested in almost all tissues and cell lines analyzed. However, this result of *ocGBP4L3* should be taken with caution due to its short length. In summary, ocGBPs differed in their endogenous mRNA expression levels.

### 2.6 Cloned ocGBP proteins are expressed in RK13 cells

**Figure 5:**
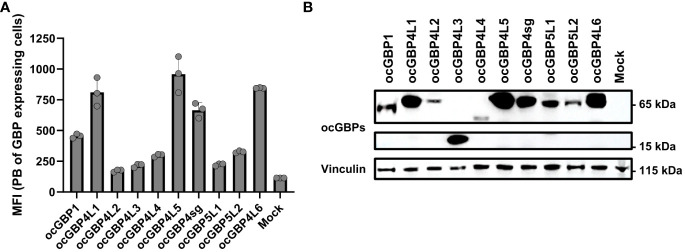
Protein expression of overexpressed rabbit GBPs in a rabbit cell line. **(A)** RK13 cells were transfected with ocGBP expression plasmids. Two days post-transfection, cells were permeabilized and protein expression was determined via flow cytometry. Shown are the mean fluorescence intensities (MFI ± SD) of HA-positive cells stained with PB-coupled antibodies (n = 3). **(B)** RK13 cells were transfected with rabbit GBP expression plasmids. Two days post-transfection, protein expression was determined using Western blot. Membranes were probed for HA tag (GBP) and Vinculin (housekeeping protein). Shown is a representative Western blot. PB, pacific blue.

To functionally characterize ocGBPs, we cloned individual ocGBPs into an expression plasmid with an HA-tag at the N-terminus. Due to the lack of ocGBP-specific antibodies, we analyzed the overexpression of ocGBPs using HA-specific antibodies. Therefore, rabbit RK13 cells were transfected with the individual ocGBPs and protein levels were determined by flow cytometry (**Figure 5A**) and Western blot (**Figure 5B**). We observed that all ocGBPs were expressed albeit at different expression levels; ocGBP4L1/4L5/4sg/4L6 were expressed to a higher level than ocGBP4L2/4L3/4L4/5L1/5L2; ocGBP1 showed an intermediate phenotype (**Figure 5**). In addition, Western blot analysis revealed the expected molecular weight for each ocGBP, ranging from 15-65 kDa (**Figure 5B**). In summary, all ocGBPs could be overexpressed at the protein level with differential expression between paralogs.

### 2.7 Varying intracellular localization patterns of ocGBPs

**Figure 6:**
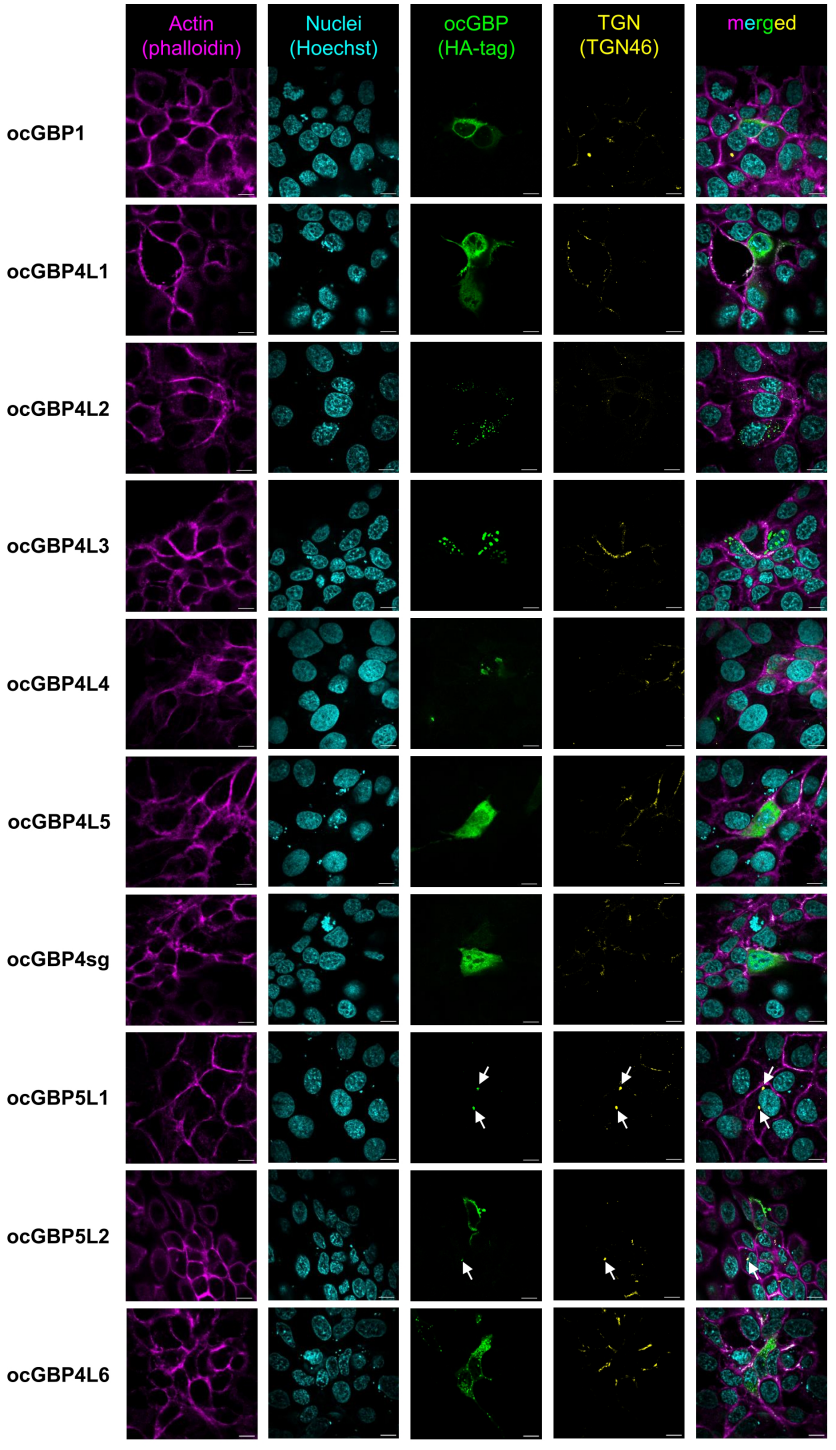
Intracellular localization of ocGBPs in RK13. RK13 cells were transfected with GBP expression plasmids. Two days post-transfection, localization was determined via immunofluorescence microscopy. The following colors were used: pink (phalloidin, actin filaments), yellow (TGN46, trans-Golgi network), indigo (Hoechst, Nucleus), green (HA-tag, GBPs). Shown are representative images out of 5 -10 imaged positions. 100x magnification, scale bars indicate 10 µm.

Since GBPs paralogs have been described to perform multiple functions (reviewed in 14) and to differ in their subcellular localization (34, 40, 41), we examined the intracellular localization of overexpressed ocGBPs in RK13 cells using confocal immunofluorescence microscopy. We observed that the rabbit paralogs localized to different intracellular compartments, with distinct patterns (**Figure 6**). ocGBP1 was distributed throughout the cytoplasm with a continuous and distinct globular localization. ocGBP4L1/4L5/4sg were evenly distributed in the cytoplasm and additionally found in the nucleus. ocGBP4L2 was localized in globular structures in the cytoplasm. ocGBP4L3/4L4 were found in distinct spots in the cytoplasm and nucleus. ocGBP4L6 was distributed in different spots in the cytoplasm and additionally found in the nucleus. We observed that ocGBP5L1 and ocGBP5L2 each co-localized with the TGN, whereas ocGBP5L2 rarely did so - it preferentially localized uniformly or polarized in the cytoplasm. In short, ocGBPs differed in their intracellular localization – some localized either uniformly and/or discretely within vesicle- or aggregate-like structures in the cytoplasm and/or nucleus and/or co-localized with the TGN.

### 2.8 Selected ocGBPs are inducible by IFNα and IFNγ

**Figure 7:**
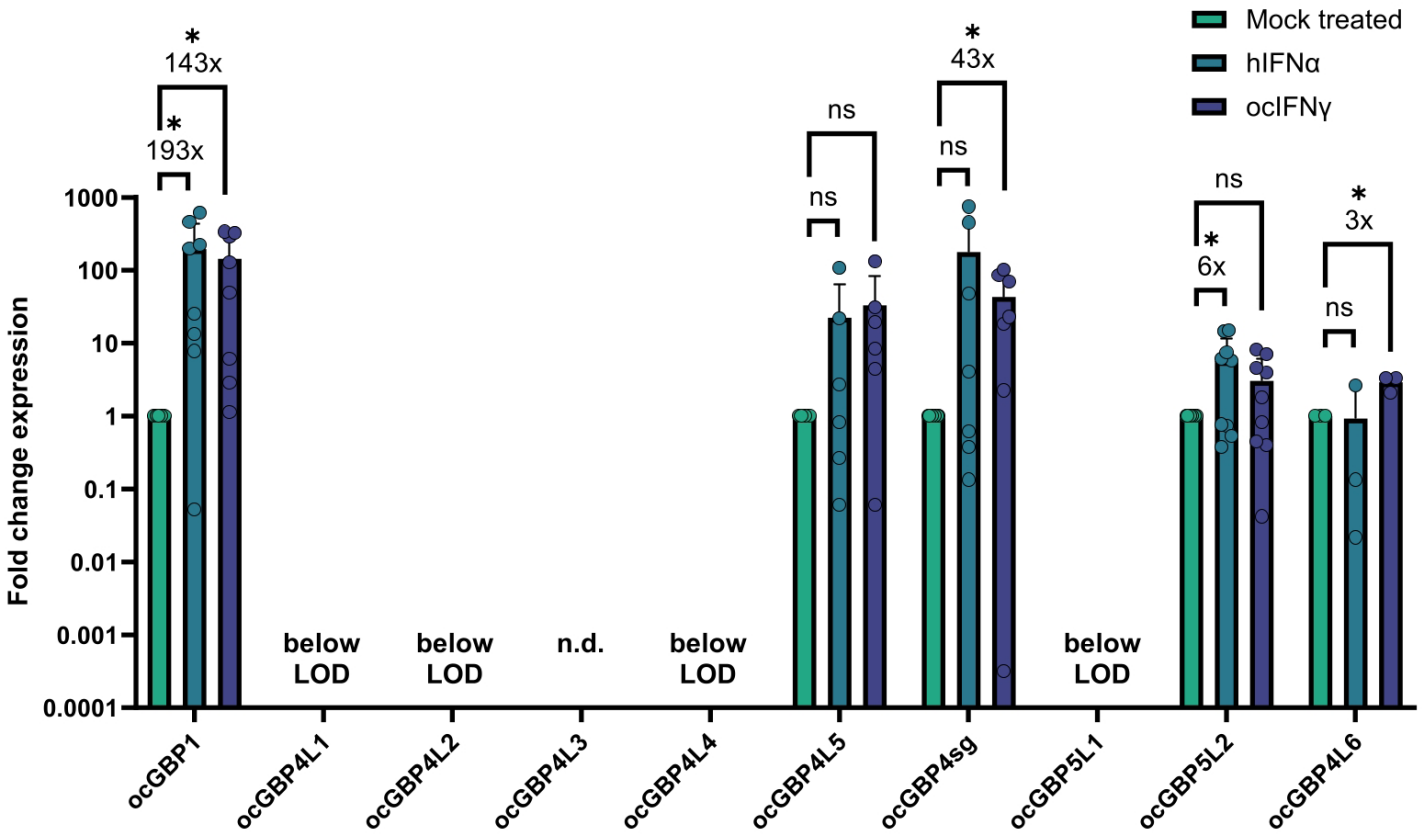
ocGBP mRNA levels in primary rabbit macrophages (Mφ) after IFN stimulation. Mφ were stimulated with human IFNα2, rabbit IFNγ, or mock-treated. mRNA levels were measured via RT-qPCR. Shown are relative expression levels that were normalized to the mock-treated samples (2^-ΔΔCt^ method). Fold change in mRNA levels compared to mock-treated are displayed (mean ± SD, 3 donors with n = 3 each). Asterisks indicate significance * p ≤ 0.05. n.s, not significant; n.d. not determined.

As a next step, we tested whether the expression of *ocGBPs* could be induced by IFN treatment. In the absence of rabbit specific reagents, we used hIFNα2 as a surrogate for ocIFNα since they share 64% aa identity. Using hIFNα2 and ocIFNγ as stimuli, we first screened two different cell lines (RK13, SIRC) and primary cells (data not shown), but IFN-inducibility was observed only in primary rabbit macrophages (**Figure 7**). We also observed that *ocGBP4L3* and *ocGBP4L5* were not IFN-inducible. For *ocGBP4L1/4L2/4L4/5L1*, IFNs did not induce them above the limit of detection (LoD). These *ocGBPs* also showed low mRNA levels in tissues, primary cells and cell lines compared to the other *ocGBPs* (**Figure 4A**). In contrast, *ocGBP1/4sg/5L2/4L6* expression was significantly induced upon IFN treatment. Specifically, the mRNA expression of *ocGBP1* was induced 194-fold and 143-fold by hIFNα2 and ocIFNγ, respectively, whereas ocIFNγ-mediated induction of *ocGBP4sg* was only 43-fold. The mRNA expression levels of *ocGBP5L2* were induced only about 6-fold by hIFNα2, and *ocGBPL6* was induced 3-fold by ocIFNγ. In summary, four out of ten *ocGBPs* were IFN-inducible in our experimental setup, suggesting that they might be involved in innate immunity as described for human and muroid GBPs.

### 2.9 Only ocGBP5L2 inhibits the activity of rabbit furin

**Figure 8:**
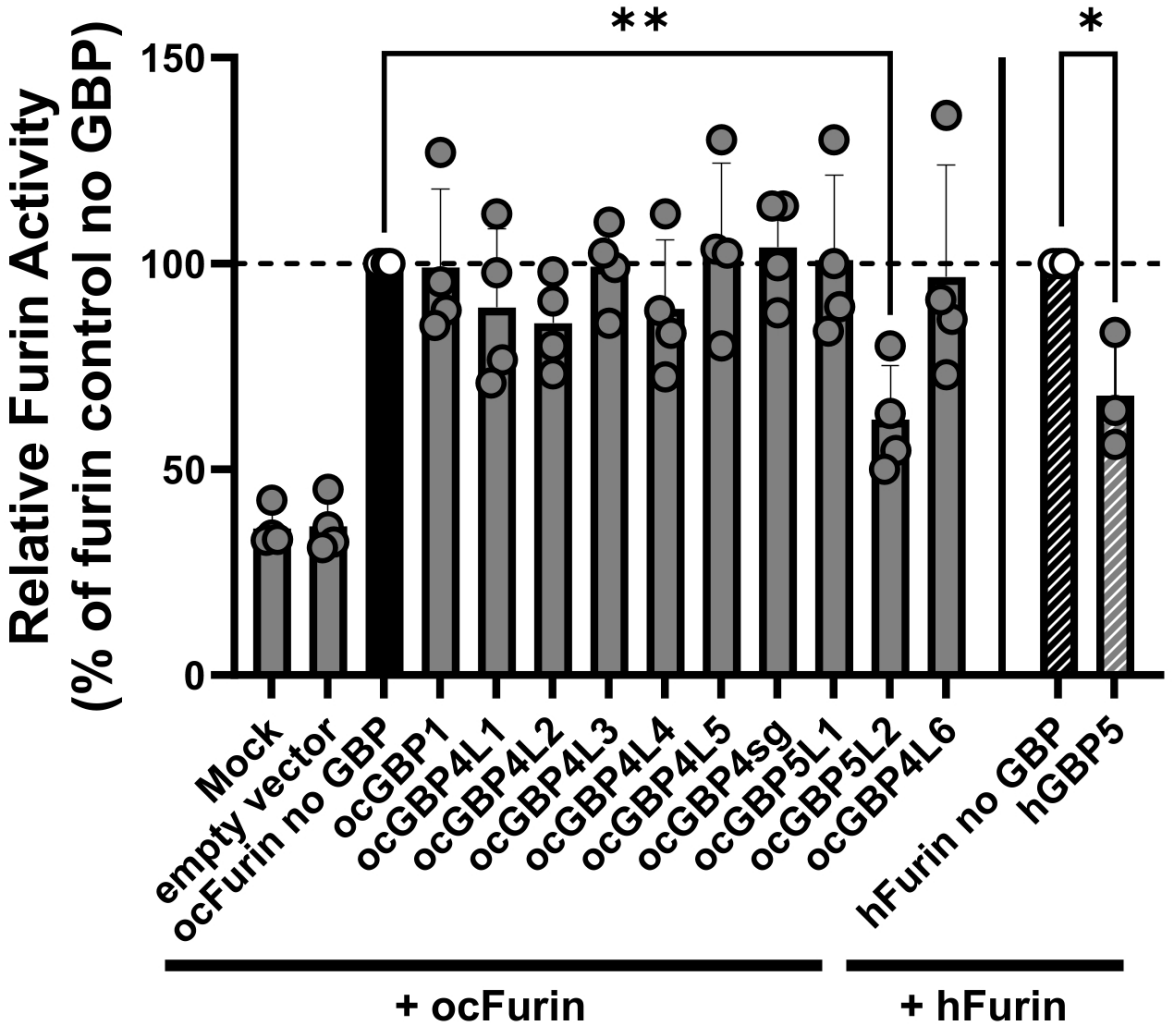
ocGBP5L2 interferes with ocFurin activity. HEK293T cells were co-transfected with furin-expression plasmids and GBP-expression plasmids. Two days post-transfection, the activity of furin secreted into the supernatant was measured after adding the AMC substrate. Shown are mean values of three independent experiments (human GBP5) or four independent experiments (rabbit GBPs). Error bars indicate SD. Asterisks indicate significance * p ≤ 0.05.** p < 0.01.

Human GBP2 and GBP5 have been shown to interfere with human furin activity (41). The cellular proprotein convertase furin has previously been described to be hijacked by several viruses for the proteolytic processing and activation of their glycoproteins (42). Consequently, hGBP2-/5-mediated furin inhibition prevents the production of fully infectious progeny virions. To determine whether ocGBPs also have the ability to affect the functionality of rabbit furin, we adapted the protocol recently developed by Braun et al. (41) to overexpress synthesized AU-1 tagged ocFurin with ocGBPs in HEK293T cells, using human furin together with hGBP5 as a positive control. Interestingly, only ocGBP5L2 inhibited ocFurin activity to a similar extent as hGBP5 for hFurin (**Figure 8**). Thus, ocGBP5L2 might be able to interfere with glycoprotein processing of various furin-dependent viruses. However, further studies need to investigate whether ocGBP5L2 inhibits viruses via supressing furin activity.

## 3 Discussion

GBPs are important players in the innate immune response against bacterial, parasitic, and viral infections. However, the breadth of their evolution and mode of action have been mainly addressed in humans and mice (reviewed in 7, 14, 43–45). Here, we expanded the current knowledge of GBP paralogs by analyzing the evolution of lagomorph GBPs and performing functional characterization of European rabbit GBPs.

*GBP3* and *7* have been exclusively found in anthropoids and primates (16). Consistent with this, we observed that these genes are absent from lagomorph genomes. Nonetheless, we found that lagomorph *GBPs* underwent a pattern of gain and loss events, similar to those described for other immunity-related genes, including GBPs (46, 47). Despite this similarity, the evolution of the lagomorph *GBP* genes, in particular in leporids, differed from that of other mammals (15–17) with a massive expansion of *GBP4*, especially in *Leporidae*. Leporids also present a unique duplication of *GBP5* compared to other mammals and lost *GBP2* (16, 17). However, *GBP2* was still present in *Ochotonidae*, suggesting conservation of *GBP* genes from the common ancestor of rodents and lagomorphs, but we also observed species-specific deletions or expansions of *GBP* genes after speciation. The resulting patterns appeared to be specific to different phylogenetic subgroups and might have been caused by host-pathogen co-evolution and/or host-specific fitness advantages against highly lethal pathogens. In addition, the unusual duplication of *GBP5* in leporids and the expansion of *GBP4* might have compensated for the loss of *ocGBP2* and *6*, respectively (**Figures 1**, **2**). Alternatively, they may have been neofunctionalized or acquired tissue-specific functions. Additionally, it has been described in humans that the recruitment of caspase-4 to the surface of *Salmonella* depends on GBP1 with the auxiliary role of GBP2 and 4 (44, 48), indicating that *Leporidae GBP4* expansion could be a compensation not only for the loss of *GBP6*, but also for the loss of *GBP2*. Comparing the evolutionary history to those of humans and mice (15–17), we could possibly identify ortholog groups, such as *GBP1, GBP4/7*-like and *GBP5*. In addition, by establishing their synteny, we clearly found similar genes flanking the GBP gene cluster as in primates and muroids (15–17). Thus, our data highlight the need for species-specific evolutionary analyses to be able to compare and translate findings from one species to another.

Similar sequences (the **Supplementary Data** GBP alignment), motifs (**Tables 1**, **2**) and tertiary structures (**Figure 3**), further backed up by their phylogenetic grouping, might imply similar functions as described for human and murine GBPs (reviewed in 7, 14, 43–45). The highly conserved G domain suggests that GTP binding and hydrolysis is an important feature of GBP proteins in general, which has already been described for other mammals (GTP hydrolysis of human GBPs reviewed in 44; GTPase domains and involvement in function reviewed in 14). Furthermore, the presence of an NLS motif (most of the ocGBPs with predicted NLS also partially localized to the nucleus, see below) and, in the same proteins, the presence of a Glu-rich domain (**Table 1**) could imply their involvement in gene regulation, although these motifs were predicted with low confidence. Several high-probability sequence motifs (**Table 2**) and putative post-translational modifications may imply tightly regulated protein expression, function, and localization, which has been described for other GBP paralogs (4, 34, 40, 49–53).

For some of the expanded genes, specifically *ocGBP4L1/4L2/4L4/5L2*, we observed that they were consistently expressed to a lower level in most tissues, primary cells and cell lines (**Figure 4**). High expression of *ocGBP4L5*/4*sg* might induce a “dosage effect” of *ocGBP4L1/4L2/4L4/5L2*, but the diversity of many ocGBP4s could still have an evolutionary advantage. They may also be tissue-specific factors that are expressed and required only in certain tissues at certain timepoints (**Figure 4**).

We observed that all overexpressed ocGBPs differed in their expression levels and yielded the expected molecular weight (**Figure 5**). Similar to the mRNA expression, there was a distinct pattern of ocGBPs with higher and lower expression levels. We saw a correlation between lower expression and localization (see below), but not with IFN inducibility. 

Varying localization of GBPs has been described in the context of human GBPs (34, 40, 41). Rabbit GBPs localized either uniformly and/or discretely within vesicular or aggregate-like structures in the cytoplasm and/or nucleus or co-localized with the TGN (**Figure 6**). We further observed that the phylogenetically coherent ocGBP4L1/4L2/4L3/4L4/4L4/4sg/4L6 and ocGBP5L1/5L2 clusters localized according to their protein expression levels (**Figure 5**), with the ocGBPs with lower protein expression forming aggregates (ocGBP4L2/4L3/4L4). We speculate that such aggregation might be harmful for homeostasis and, therefore, locally restricted. ocGBP5L1 and rarely ocGBP5L2 co-localized with the TGN (**Figure 6**) as described for hGBP5, for which the localization was suggested to be required for its antiviral activity (41). This is not expected since ocGBP5L1, unlike ocGBP5L2, does not have a CaaX motif (**Table 2**). Of note, for ocGBP5L2, we rarely observed this co-localization, as we more often observed a uniform localization to the cytoplasm or a polarized localization in the cytoplasm (**Figure 6**). In contrast to human GBP5 (41), ocGBP5L1 co-localized with the Golgi, which suggests that the prenylation is not the only determinant and other described modifications, such as N-myristoylation present in both ocGBP5s, could also play a role (**Table 2**). Since ocGBP5L2 only rarely localized to the TGN, we speculate that ocGBP5L1 and ocGBP5L2 may form heterodimers as described for other GBPs (34, 40) and thus increase the affinity to bind to the TGN for antiviral activity.

We found that the mRNA levels of ocGBP1, ocGBP4sg, ocGBP5L2 and ocGBP4L6 were significantly induced by IFN treatment in primary rabbit macrophages (**Figure 7**). In addition, ocGBP5L2 inhibited the activity of ocFurin (**Figure 8**). This would suggest that despite their genetic diversity compared to muroid and human GBPs, they play a similar role in immune responses as those described for mouse and human GBPs. The cause of the differentially induced expression of GBPs by IFNs could be their involvement in different functions in the innate immune response, as observed for human GBPs with specific paralogs involved in the response to different (classes of) pathogens or in inflammatory and cancer pathways (reviewed in 7, 14, 43–45). For hGBPs, one explanatory approach is the difference in 5’ regulatory elements for IFN-dependent transactivators between the different paralogs obtained from CHIP-seq ENCODE data (43).

We observed that the different rabbit paralog groups (ocGBP1 induced by both IFN, ocGBP4 by IFNγ and ocGBP5 by IFNα) have distinct group-specific structural features (**Tables 1**, **2** and **Figure 3**). This could imply a similar but distinct function for the different paralog groups. This is contradicted by the different IFN induction within the groups (**Figure 7**), but could be explained by the loss/gain of an IFN-dependent 5’ regulatory element in the gene duplication process, so that these genes may have acquired new or additional functions, or may still be functional at constitutionally lower levels of expression. Despite the structural similarity to ocGBP5L1, only ocGBP5L2 inhibited furin activity (**Figure 8**). Therefore, the CaaX motif may be essential for furin inhibition.

In conclusion, our work adds valuable information to the evolution of ocGBPs and their characteristics, and implicates implicates a role of ocGBPs in innate immunity, which needs to be evaluated in future studies.

## 4 Materials and methods

### 4.1 Synteny

Syntenic positions and transcription orientations of lagomorph *GBP* were inferred by visual inspection of their genomes in publicly available databases NCBI (https://www.ncbi.nlm.nih.gov/genome/gdv/) and Ensembl (https://www.ensembl.org/index.html).

### 4.2 Phylogeny

Gene sequences annotated as *GBP* were retrieved in the timeframe March to August 2021 from publicly available databases. A total of 202 sequences were retrieved (see **Supplementary Table 1**): 19 sequences were retrieved from three different lagomorph species (*Ochotona princeps*, *Ochotona curzoniae* and *Oryctolagus cuniculus*), 41 sequences from 6 species of primate origin, including *Homo sapiens*; 123 sequences from 12 rodent species; *Tupaia glis* (5 sequences), *Loxodonta africana* (7 sequences) and *Gallus gallus* (7 sequences), the latter of which was used as outgroup. To ensure that all *GBP* sequences from all the species were included, a subsequent BLAST analysis was performed. Sequences that did not encode a functional protein or presented partial mRNA sequences were excluded from the analysis (accession numbers available in **Supplementary Table 1**). Alignment of the sequences was performed in BioEdit software (54) using the Clustal ω method (55) followed by visual inspection and correction. Alignment of GBP protein sequences can be found in the **Supplementary Data** GBP alignment. In addition, the alignment was screened for gene conversion/recombination using GARD (27). Phylogenetic relationships were inferred in MEGAX (56) using the Maximum Likelihood (ML) method and the Jones-Taylor-Thornton matrix-based substitution model + G + I as determined by MEGAX (57). To assess the robustness of the tree branches, 1000 bootstrap replicates were used. The trees were drawn to scale, with branch lengths measured in the number of substitutions per site. All positions with less than 95% site coverage were eliminated, i.e., fewer than 5% alignment gaps, missing data, and ambiguous bases were allowed at any position (partial deletion option).

### 4.3 ProSite Scan

The ProSite Scan tool was used to identify (functional) protein sequence motifs (31–33). Protein sequences of the ocGBPs were included to scan them against the PROSITE collection of motifs. The scan was performed at high sensitivity.

### 4.4 Protein structure modeling with AlphaFold

For structure prediction, ChimeraX (https://www.rbvi.ucsf.edu/chimerax) (58) was used with the structure prediction AlphaFold tool with the corresponding ocGBP protein sequences. Computations were performed on Google Colab using ColabFold, an open source, optimized version of AlphaFold 2 (59). The resulting prediction models were visualized using ChimeraX (58).

### 4.5 Rabbit organ and serum preparation

Four 36-to 40-days old female New Zealand white rabbits (*Oryctolagus cuniculus*) were ordered from Charles River (France) and housed for an additional acclimation week prior to organ removal in a specific pathogen-free (SPF)-barrier. The laboratory conditions and husbandry of the animals were identical to a recently published study (60). They were euthanized by slow intravenous injection of a lethal dose of sodium-pentobarbital (100 mg/kg Narcoren, Boehringer Ingelheim, Ingelheim am Rhein, Germany). The following organs were collected: spleen, liver, heart, appendix, stomach, kidney, brain, colon, small intestine, lungs, thymus and uterus. The organs were frozen in liquid nitrogen and homogenized to frozen powder, which was stored at -80°C prior further processing. For the preparation of rabbit serum, rabbit blood was collected from the heart and incubated at 37°C for 30 min, followed by 30 min on ice. The blood was then centrifuged at 12 000 g for 10 min. The experiments have been approved by the institutional ethical review committee (LMU Munich, Biomedical Center, Core facility animal models) and are in accordance with the local government authorities Az.5.1-5682 (LMU/BMC/CAM) as well as European (RL2010/63EU) and German animal welfare legislation.

### 4.6 Preparation of splenocytes and macrophage and T cell differentiation

Rabbit splenocytes were prepared by mashing the spleens through a 40 µm cell strainer (LABSOLUTE) in 1x PBS until only rigid scaffolds (capsules) were left. The cells were subsequently pelleted for 5 min at 500 g and the remaining red blood cells were lysed with 4 ml ACK lysis buffer (8.29 g/l NH_4_CL (Carl Roth), 1 g/l KHCO_3_ (Carl Roth), 0.0367 g EDTA (CHEMSOLUTE)) for 5 min at room temperature (RT) and washed in 1x PBS for 5 min at 500 g. The procedure was repeated until lysis was complete. Splenocytes were cultivated in RPMI 1640 GlutaMAX™ (Gibco) supplemented with 10% (v/v) FCS (Sigma-Aldrich) and 1% (v/v) Penicillin-Streptomycin (10,000 units Penicillin and 10 mg Streptomycin per ml, Sigma-Aldrich) at standard conditions (37°C; 5% CO_2_; 90% humidity). For T cell differentiation, splenocytes were maintained at 2 x 10^6^ cells/ml with 100 U/ml human recombinant IL-2 (Biomol #50442) and 5.0 µg/ml Concanavalin A (Sigma-Aldrich #C2010) for four days and then only cultivated in IL-2 containing medium. For the differentiation of rabbit macrophages, 2 x 10^6^ cells/ml rabbit splenocytes were seeded into 12-well plates with 2% (*v/v*) rabbit serum for one week until heterogeneous differentiation could be observed.

### 4.7 Cell culture cell lines

SIRC (Cornea, ATCC CCL-60), RAB-9 (Skin, ATCC CRL-1414), RK13 (Kidney, ATCC CCL-37) and RL-33 (Lung, tebu-bio JCRB0131) cell lines were cultured in monolayers in MEM GlutaMAX™ (Gibco) supplemented with 10% heat-inactivated fetal calf serum (FCS, Sigma Aldrich) and 1% Penicillin/Streptomycin (P/S, Sigma Aldrich). 55D1 (B-cell line, (61); kind gift of Dr. Katherine L. Knight) and RL-5 (T-cell line; 62) were cultured in RPMI 1640 GlutaMAX™ Medium (Gibco) supplemented with 10% FCS (Sigma Aldrich) and 1% P/S (Sigma Aldrich). HEK293T cells (Kidney, human, DSMZ ACC 635) were cultured in monolayers in DMEM GlutaMAX™ (Gibco) supplemented with 10% FCS (Sigma Aldrich) and 1% P/S (Sigma Aldrich). All cells were cultured at 37°C and 5% CO_2_ and 90% humidity.

### 4.8 RT-qPCR

RNA was extracted from the samples using NucleoZol (Macherey-Nagel); the remaining genomic DNA was digested using TURBO DNA-free™ Kit (Invitrogen) and cDNA was subsequently generated using the High-Capacity RNA-to-cDNA™ Kit (Applied Biosystems). All three steps were conducted according to the manufacturer’s instructions. One ng/µl cDNA was prepared for cell lines and primary cells and 10 ng/µl for tissues samples. Analysis of gene expression was performed using PowerUp™ SYBR™ Green Master Mix (Applied Biosystems) with a Quantstudio 3 Real-Time PCR system (Applied Biosystems). The reactions were set up using 5 µl PowerUp™ SYBR™ Green Master Mix, 2 µl nuclease-free water, 0.5 µl of 10 µM forward and reverse primer each (**Table 3**) and 2 µl of respective cDNA to a total reaction volume of 10 µl. The following thermal cycling conditions were used: hold stages at 50°C for 2 min and at 95°C for 2 min, 40 cycles with denaturation at 95°C for 1 s and annealing/elongation at 60°C for 30 s. Finally, the melting curve was performed with 95°C for 1 s and 60°C for 20 s with a rate of 0.1°C/s from 60°C to 95°C. Ct values were used to determine gene expression in relation to the reference gene. Optimal qPCR primers were designed using primer3 (https://primer3.ut.ee/) (80-120 bp amplicon length, 20 bp optimal length and 60°C optimal T_m_, **Table 1**) (63, 64). One primer of each primer pair was spanning an exon-exon junction. Results were analyzed as ΔCt = Ct (ocGBP) – Ct (Actin β (ActB)).

**Table 3:** RT-qPCR primers for lagomorph gene expression analyses.

	Accession Number	Primer	Sequence 5’-3’
**ocGBP1**	XM_002715873.3	q_ocGBP1_f	AGCAAGGGGTCTTTTCTAAACC
		q_ocGBP1_r	TCTTCAGCCTGTATCCCTTTCC
**ocGBP4 L1**	XM_008264927.2	q_ocGBP4_L1_f	CGAAAGAAACTTACCGACACCAT
		q_ocGBP4_L1_r	CGAAAGCCGCCTAAGTTCAG
**ocGBP4 L2**	XM_017346115.1	q_2_ocGBP4_L2_f	CCTGTAGTAGTAGTGGCCATTGT
		q_2_ocGBP4_L2_r	CAGAGGGAAGCCATGTTTCTG
**ocGBP4 L3**	XM_017345575.1	q_3_ocGBP4_L3_f	TCTTAACCAGATATCTCAGCCTGT
		q_3_ocGBP4_L3_r	GGGAAGCCATGTTTCTGTCCT
**ocGBP4 L4**	XM_017346109.1	q_2_ocGBP4_L4_f	GCACAAGCTGAAGGCTCAAA
		q_2_ocGBP4_L4_r	TCTCTTCTGTTAGCCGCTTGA
**ocGBP4 L5**	XM_002715512.3	q_2_ocGBP4_L5_f	AGAAGATGGAGCGGGAAAGG
		q_2_ocGBP4_L5_r	AGCATTTCTTCTTGGACCTTCAG
**ocGBP4sg**	XM_008264924.2	q_2_ocGBP4_sg_f	AGCACAAGCTGAAGGTTCAAA
		q_2_ocGBP4_sg_r	GCTGCCATATCTTCTGTTATCCG
**ocGBP5 L1**	XM_008265608.2	q_2_ocGBP5_L1_f	AGAGGTGTGGCAAATGGAGA
		q_2_ocGBP5_L1_r	ATTGCAGCCTCCTCCTGG
**ocGBP5 L2**	XM_002715513.3	q_3_ocGBP5_L2_f	AGAGGTGCGACAAATGGAGA
		q_3_ocGBP5_L2_r	CTCTGAGCCTCTTCCTGGAG
**ocGBP4L6**	XM_008264918.2	q_2_ocGBP4L6_f	CCAGGAGAACATCACCCAGT
		q_2_ocGBP4L6_r	AGCAGGTCTTCTTGGATCTTCA
**Actin beta (ActB)**	NM_001101683.1	ActB_L_f	TCCTGGGCATGGAGTCGT
**reference gene**		ActB_L_r	GTGTTGGCGTACAGGTCCT

### 4.9 BLAST analysis for rabbit transcriptome

Rabbit transcriptome was generated as part of the rabbit genome paper (39) and the deposited data were analysed using the BLAST tool from NCBI (https://blast.ncbi.nlm.nih.gov/Blast.cgi).

### 4.10 Cloning of ocGBPs in expression plasmid

Template cDNAs for amplification of the rabbit *GBPs* were prepared as described above. pCG vector was used as backbone template. Rabbit *GBPs* were amplified using the primer pairs in **Table 4** by PCR (**Tables 5**, **6**) with the cDNAs as template. An HA-tag for detection purposes was added at the N-terminus. Since ocGBP5L1 could not be amplified from rabbit cDNAs, it was ordered from Twist Bioscience (accession number: XM_002715873.3). The final pCG-HA-GBP plasmids were obtained via Gibson assembly using NEBuilder® HiFi DNA Assembly Master Mix (NEB) according to the manufacturer’s protocol and sequence-verified using Sanger sequencing.

**Table 4:** PCR primers to clone rabbit GBPs.

Primer	Sequence 5'-3'
pCG_amp_f	ACGCGTCGGATCCTGAGAAC
**pCG_amp_HA_r**	AGCGTAATCTGGAACATCGTATGGGTACATTCTAGAAGGCCTACGCGCTTC
**gib_3.1_pCG_rbGBP1_f**	GTACCCATACGATGTTCCAGATTACGCTATGACCTCAGAGATCCACATG
**gib_3.1_pCG_rbGBP1_r**	CTGAAGTTCTCAGGATCCGACGCGTTTAGCTTATAACACATCTTCTCCTTGG
**gib_3.4L1_pCG_rbGBP4_L1_f**	GTACCCATACGATGTTCCAGATTACGCTATGGCAACCGAATTTATGAATG
**gib_3.4L1_pCG_rbGBP4_L1_r**	CTGAAGTTCTCAGGATCCGACGCGTCTATTTAATTTGTGAACTGATAAATCGC
**gib_3.4L2_pCG_rbGBP4_L2_f**	GTACCCATACGATGTTCCAGATTACGCTATGGCAACTGAATTCACCATG
**gib_3.4L2_pCG_rbGBP4_L2_r**	CTGAAGTTCTCAGGATCCGACGCGTCTATGCAGTTGTTAAAGTCTGGT
**gib_3.4L3_pCG_rbGBP4_L3_f**	GTACCCATACGATGTTCCAGATTACGCTATGGCAACTAATATCACCATGAAG
**gib_3.4L3_pCG_rbGBP4_L3_r**	CTGAAGTTCTCAGGATCCGACGCGTTTAAACTGTAAGAGCACAGTTGAG
**gib_3.4L4_pCG_rbGBP4_L4_f**	GTACCCATACGATGTTCCAGATTACGCTATGGCGACTGATATCACC
**gib_3.4L4_pCG_rbGBP4_L4_r**	CTGAAGTTCTCAGGATCCGACGCGTCTATAACTTTCTTAACAGCCTTGA
**gib_3.4L5_pCG_rbGBP4_L5_f**	GTACCCATACGATGTTCCAGATTACGCTATGGCAACTGATATCACCATG
**gib_3.4L5_pCG_rbGBP4_L5_r**	CTGAAGTTCTCAGGATCCGACGCGTTCAGTCTTTAGATTTTGAACCAAG
**gib_3.4sg_pCG_rbGBP4sg_f**	GTACCCATACGATGTTCCAGATTACGCTATGGCAACTGATACTACCATG
**gib_3.4sg_pCG_rbGBP4sg_r**	CTGAAGTTCTCAGGATCCGACGCGTCTATAAAATTCTTCGACTCAGTCTTAAC
**gib_3.5L1_pCG_rbGBP5_L1_f**	GTACCCATACGATGTTCCAGATTACGCTATGGCCTCGGAGATCCTC
**gib_3.5L1_pCG_rbGBP5_L1_r**	CTGAAGTTCTCAGGATCCGACGCGTTTATCTCTTTGGTGAAAAGAAAGTTCCA
**gib_3.5L2_pCG_rbGBP_ L2_f**	GTACCCATACGATGTTCCAGATTACGCTATGGCCTTGGAGATCCTC
**gib_3.5L2_pCG_rbGBP_ L2_r**	CTGAAGTTCTCAGGATCCGACGCGTTTAGAGTAAGATGCAATCATCATTTGG
**gib_3.7_pCG_rbGBP4L6_f**	GTACCCATACGATGTTCCAGATTACGCTATGGACACCACAAATCCTG
**gib_3.7_pCG_rbGBP4L6_r**	CTGAAGTTCTCAGGATCCGACGCGTCTATTTTATTTGTGTGCTCAACATTTTC

**Table 5:** PCR reaction components for GBP cloning.

COMPONENT	VOLUME (µl)	FINAL CONCENTRATION
**5X Phusion™ HF Buffer (Thermo Scientific)**	10 µl	1X
**10 mM dNTPs (Thermo Scientific)**	1 µl	200 µM
**10 µM Forward Primer (Eurofins)**	2.5 µl	0.5 µM
**10 µM Reverse Primer (Eurofins)**	2.5 µl	0.5 µM
**Template DNA**	1 pg–10 ng (plasmid or viral); 50 ng– 250 ng (genomic)	< 1,000 ng
**Phusion™ High–Fidelity DNA Polymerase (Thermo Scientific)**	0.5 µl	0.02 U/µl
**Nuclease-Free Water**	add to 50 µl	

**Table 6:** PCR thermocycler program to clone rabbit GBPs.

Temperature [°C]	Time [s]	cycles
98	60	
98	10	
Ta*	30	35 x cycles
72	75	
72	360	
10	hold	

*optimal annealing temperature of the respective primer pairs.

### 4.11 Transfection for protein overexpression

For heterologous expression of the ocGBPs, 1.2 x 10^5^ RK13 cells, seeded one day prior to transfection in a 12-well plate, were transfected with 1.5 µg of each pCG-HA-ocGBP plasmid, respectively, using TurboFect transfection reagent (Thermo Fisher) in 100 µl of unsupplemented medium, according to manufacturer’s instructions. The mixture was incubated at RT for 45 min and then added dropwise to the cells. Cells were incubated at 37°C, 5% CO_2_ and 90% humidity and expression was analyzed two days post-transfection.

### 4.12 Flow cytometric analyses

For flow cytometry detection of ocGBP expression, transfected cells were intracellularly stained for the HA-tag. Briefly, detached RK13 cells were fixed with 100 μL pre-warmed PFA (4% in 1x PBS, AppliChem) at RT for 10 min. The cells were washed once with 1 x PBS (Sigma Aldrich), and the supernatant was aspirated. For permeabilization, 100 µl of pre-cooled BD Phosflow Perm Buffer III was added to each well and incubated for 2 min on ice. Cells were washed twice with 1 x PBS, then resuspended and stained with 50 µl of Pacific Blue™ anti-HA.11 epitope tag antibody (1:100; #901526, Biolegend) in staining buffer (1x PBS (pH 7.2), 1% (*v/v*) FCS (Sigma-Aldrich), 0.09% NaN_3_(Carl Roth)) at RT for 45 min in the dark. Cells were washed once with staining buffer and then resuspended in 200 µl of staining buffer for subsequent analysis using the BD FACSLyric™ Flow Cytometer. Data were analyzed using the FlowJo software.

### 4.13 Western blot

Cells were lysed in 50 µl Hunt lysis buffer (20 mM Tris-HCl pH 8.0 (Carl Roth), 100 mM sodium chloride (Carl Roth), 1 mM EDTA (CHEMSOLUTE), 0.5% NP-40 (AppliChem) containing 1 x cOmplete protease inhibitor cocktail (Roche)). Lysates were cleared by centrifugation at 20,000 *g* and 4°C for 20 min. Protein concentration was quantified with the Quick Start Bradford 1 x and Quick Start Bovine Serum Albumin Standard Set (both Bio-Rad) according to the manufacturer’s instructions using CLARIOstar (BMG Labtech). 4 x Laemmli buffer (Bio-Rad) supplemented with 50 mM DTT (Carl Roth) was added to a final concentration of 1 x to the samples, which were then denatured at 95°C for 5 min. NuPage 4-12% Bis-Tris gels were used (Invitrogen). 15 µg total protein was loaded per sample. Gel electrophoresis was performed in 1 x MOPS-SDS running buffer using a Mini Gel Tank (both Invitrogen) at 100 V for 90 min. Afterwards, the proteins were transferred onto nitrocellulose membranes (0.45 μm, Bio-Rad) in 1 x Tris-Glycine Transfer Buffer (25 mM Trizma base, 192 mM Glycine, 20% (*v/v*) methanol (CHEMSOLUTE)) using the Mini Blot Module (Invitrogen) at constant voltage (14 V, 75 min). Membranes were blocked in TBS-T (20 mM Tris, 150 mM NaCl, 0.1% (*v/v*) Tween 20 (Carl Roth) containing 5% (*w/v*) powdered milk (Carl Roth). Proteins were stained with the following primary antibodies overnight: HA tag polyclonal antibody (SG77, #71-5500, 1:250, Invitrogen) and Vinculin recombinant rabbit monoclonal antibody (42H89L44, #700062, 1:1000, Invitrogen). The following day, membranes were washed three times with 1 x TBS-T, incubated for 1 h at RT in horseradish peroxidase (HRP)-coupled secondary antibody peroxidase AffiniPure goat anti-rabbit IgG (H+L) (#AB_2313567, Jackson Immunoresearch), diluted 1:10000 in 1 x TBS-T containing 5% (*w/v*) powdered milk. After three washing steps in 1 x TBS-T, blots were visualized with the SuperSignal™ West Femto Maximum Sensitivity Substrate (Thermo Scientific) according to the manufacturer´s instruction using the FUSION FX (Vilber).

### 4.14 Immunofluorescence

RK13 cell were seeded on 13 mm glass cover slips (VWR) and transfected as above. Transfected cells were fixed with 100 µl pre-warmed PFA (4% in 1x PBS, AppliChem) at RT for 15 min, permeabilized with 100 µl 0.1% Triton X-100 (Carl Roth) in 1x PBS for 5 min at RT and blocked with 1 x PBS with 2% (*w/v*) BSA (Carl Roth) for 30 min at RT. First, the actin filaments were stained using phalloidin Atto-647N (10 µM in MeOH, #AD647N-81, 1:60, ATTO-TEC) in 1x PBS for 30 min at RT. For TGN staining, primary antibody sheep anti human TGN46 (#AHP500G, 1:1000, Bio-Rad) was first incubated in 1 x PBS with 2% (*w/v*) BSA for 60 min at RT. Subsequently, cells were stained with secondary antibody donkey anti-sheep IgG (H+L) cross-adsorbed secondary antibody Alexa Fluor 568 (#A-21099, 1:2000, Invitrogen) and directly-coupled anti-HA.11 epitope tag antibody Alexa Fluor 488 (#901514, 1:1000, Biolegend) in 1 x PBS with 2% (*w/v*) BSA for 1 h at RT. Lastly, nuclei were counterstained with 1 µg/ml Hoechst 33342 solution (#62249, Thermo Scientific) for 15 min at RT. Cells were then washed using Millipore water to get rid of salts. Cover slips were mounted with ProLong Gold Antifade Mountant (Invitrogen) on microscope slides (Carl Roth) and dried for 24 h at RT before microscopy analyses were performed using the Yokogawa Spinning Disk Field Scanning Confocal System CSU-W1 (Nikon) with 100 x magnification and following filters: Filter block 1: EX 387/11 EM 416 LP, Filter block 2 EX 469/35 EM BA 525/39, Filter block 3: EX 559/34 EM 639/69, Filter block 4: EX 628/40 EM 692/40.

### 4.15 Interferon stimulation

RK13, SIRC and M0 macrophages (prepared as described above) were stimulated with either with 20 ng/ml human IFNα2 (#592702, Biolegend), rabbit IFNγ (#RP0136U-005, Kingfisher Biotech) or mock-treated, and harvested 24 h post stimulation. Cells were prepared as described above for RT-qPCR, which was conducted using the same primers and protocol. Results were analyzed using 2^-ΔΔCt^ method.

### 4.16 Furin activity measurement

ocFurin was synthesized by basegene (Leiden, Netherlands) and subcloned into the pCG C-AU-1IRES BFP vector (41). To determine furin activity in HEK293T cells, the assay was essentially performed as previously described (41) by co-transfecting cells in a 96-well cell culture plate with 50 ng furin-expressing plasmids and 75 ng GBP-expressing plasmids. Two days post-transfection, 20 μL of cell culture supernatant was incubated with the Pyr-Arg-Thr-Lys-Arg-7-Amido-4-methylcoumarin (AMC) substrate (1 nmol), and furin activity was determined for 5 h with an interval of 2 min using a Cytation3 imaging reader (355 nm excitation and 460 nm emission).

### 4.17 Statistics

Statistical analyses were performed using GraphPad Prism 9 using Students’ t-test.

## Data availability statement

The original contributions presented in the study are included in the article/**Supplementary Material**; further inquiries can be directed to the corresponding authors.

## Ethics statement

The animal study was approved by the local government authorities Az.5.1-5682 (LMU/BMC/CAM) as well as European (RL2010/63EU) and German animal welfare legislation. The study was conducted in accordance with the local legislation and institutional requirements.

## Author contributions

LS: Conceptualization, Data curation, Formal analysis, Investigation, Methodology, Writing – original draft, Writing – review & editing. JVCR: Conceptualization, Data curation, Formal analysis, Investigation, Writing – original draft, Writing – review & editing. SF: Investigation, Formal analysis, Writing – review & editing. AB: Investigation, Writing – review & editing. MS: Investigation, Formal analysis, Writing – review & editing. MP: Investigation, Formal analysis, Writing – review & editing. RL: Formal analysis, Supervision, Writing – review & editing. BM: Investigation, Writing - review & editing. DM: Resources, Writing – review & editing. LP: Resources, Writing – review & editing. AP: Data curation, Writing – review & editing. JM: Data curation, Resources, Writing – review & editing. JM-F: Data curation, Resources, Writing – review & editing. BP: Resources, Supervision, Writing – review & editing. PE: Funding acquisition, Supervision, Writing – review & editing. DS: Funding acquisition, Supervision, Writing – review & editing. JA: Funding acquisition, Supervision, Writing – review & editing. H-MB: Conceptualization, Funding acquisition, Supervision, Writing – original draft, Writing – review & editing.
